# Impact of Calcium and Magnesium in Groundwater and Drinking Water on the Health of Inhabitants of the Slovak Republic

**DOI:** 10.3390/ijerph14030278

**Published:** 2017-03-08

**Authors:** Stanislav Rapant, Veronika Cvečková, Katarína Fajčíková, Darina Sedláková, Beáta Stehlíková

**Affiliations:** 1 State Geological Institute of Dionyz Stur, Mlynská Dolina 1, 817 04 Bratislava, Slovak Republic; veronika.cveckova@geology.sk (V.C.); katarina.fajcikova@geology.sk (K.F.); 2 WHO Country Office in the Slovak Republic, Limbová 2, 837 52 Bratislava, Slovak Republic; sedlakovad@who.int; 3 Faculty of Economics and Business, Paneuropean University, Tematínska 10, 851 05 Bratislava, Slovak Republic; stehlikovab@gmail.com

**Keywords:** groundwater, health status of inhabitants, Ca, Mg, mortality from cardiovascular, oncological, gastrointestinal and respiratory diseases

## Abstract

This work aims to evaluate the impact of the chemical composition of groundwater/drinking water on the health of inhabitants of the Slovak Republic. Primary data consists of 20,339 chemical analyses of groundwater (34 chemical elements and compounds) and data on the health of the Slovak population expressed in the form of health indicators (HI). Fourteen HIs were evaluated including life expectancy, potential years of lost life, relative/standardized mortality for cardiovascular and oncological diseases, and diseases of the gastrointestinal and respiratory systems. The chemical and health data were expressed as the mean values for each of the 2883 Slovak municipalities. Artificial neural network (ANN) was the method used for environmental and health data analysis. The most significant relationship between HI and chemical composition of groundwater was documented as Ca + Mg (mmol·L^−1^), Ca and Mg. The following limit values were set for these most significant groundwater chemical parameters: Ca + Mg 2.9–6.1 mmol·L^−1^, Ca 78–155 mg·L^−1^ and Mg 28–54 mg·L^−1^. At these concentration ranges, the health of the Slovak population is the most favorable and the life expectancy is the highest. These limit values are about twice as high in comparison to the current Slovak valid guideline values for drinking water.

## 1. Introduction

With respect to human health, chemical elements in the environment (i.e., contained within the water, soil, and air) can be present in amounts that may be deficient or excessive. Each chemical element can be thought of as a “medicine” or a “poison”; it depends on the dose (Paracelsus). There are three main exposure routes for chemical elements to reach the human organism: ingestion, inhalation, and dermal contact [1]. In a natural, non-contaminated environment, ingestion of food and water is considered to be the main exposure route of chemical elements to humans. Chemical elements soluble in drinking water occur mainly in the ionic form, and are generally directly bioavailable to humans. On the other hand, chemical elements in soils may enter the crops only if dissolved in soil water (moisture) and thus be ingested through the food chain. The input of chemical elements from soils to humans through the food chain, and their potential manifestation into health effects depends on many factors, mainly their concentration, bioavailability, and speciation [2,3,4,5,6,7].

At present, in many regions of the world, people consume foodstuffs that are of global origin. The majority of the food consumed comes from various regions of the world, and their chemical compositions reflect the chemistry of the soil and water from where they come from. Only a small amount of the food consumed, mainly vegetables and fruits, is of local origin, and its chemical composition reflects the local geological structure and therefore local geochemical background.

In the case of drinking water, the situation is different. People use water for drinking and cooking from the same source, having the same chemical composition for long periods of time, often over the course of their lifetime, or until they move to another place. Thus, the variability of the chemical composition of the water that people consume is not as high as that of the foods they consume. In the case of a deficit in or excess of certain chemical elements that are essential to human health, health effects can occur with long-term consumption of that water. Several studies dealing with the relationship of Ca and Mg in drinking water and the mortality from cardiovascular diseases report that a one-year exposure is a sufficient period to manifest into significant health effects [8,9]. However, for cancer it may take a much longer period of time.

The health effects of classic contaminants found in drinking water, e.g., potentially toxic elements (As, Cd, Cr, etc.) and compounds such as nitrates or nitrites, are well known and well documented [10,11,12,13,14,15]. Due to their known adverse health effects, these contaminants are strictly limited in drinking water guidelines and regulations. However, the influence of other, mainly essential elements (e.g., Ca, Mg, K) on human health is not currently well recognized, and that is why they are not limited in guidelines such as the World Health Organization (WHO) drinking water guideline [16]. In other cases, these essential elements are only limited in the form of recommended values, such as in the Slovak guideline for water used for human consumption [17]. There are many publications documenting increased incidence and mortality rates for cardiovascular diseases associated with a deficit of Ca and Mg in drinking water [18,19,20,21,22,23,24]. Several studies can be found that link these deficits to increased mortality from oncological diseases as well [25,26,27,28,29,30]. As of yet, there is no documentation of the influence of Ca and Mg deficiency in drinking water on the respiratory and gastrointestinal systems.

This paper deals with the evaluation of a relatively wide range of chemical elements in the groundwater and drinking water consumed in the Slovak Republic in relation to the health of the population. Various indicators of health and demographic growth of the population are linked to the contents of chemical elements and compounds in the groundwater, known as environmental indicators. Therefore, we evaluate the impact of groundwater originating from various geological environments and of various geneses reflected in variable chemical composition on the most common causes of deaths. The health issues considered include cardiovascular and oncological mortality, mortality from diseases of the gastrointestinal tract and respiratory system, and life expectancy. We used several mathematical and statistical methods (i.e., Pearson and Spearman correlations and artificial neural network) for linking data on the chemical composition of groundwater with various causes of death. The method of neural network was used for the derivation of limit values for chemical elements in groundwater to define levels at which the health of the population is most favorable.

## 2. Materials and Methods

### 2.1. Environmental Indicators

Environmental indicators (EI) represent chemical elements, chemical compounds, and chemical parameters that are analyzed, measured, and monitored in the environment, which can affect biota or humans [31]. In this work we evaluated 34 EI, mainly inorganic components in groundwater including all common macroelements, trace elements, and basic parameters of natural radioactivity. We did not assess organic pollutants because there is no available data collected at the required density across the entire territory of the Slovak Republic.

The data source for groundwater chemical composition comes from national geochemical databases for Slovak groundwater, including the analyses carried out since 1991, when the modern environmental geochemical mapping of the Slovak Republic started [32,33]. Our database includes virtually all sources of groundwater used for bulk supply of drinking water for the country. The total number of chemical analyses of groundwater was 20,339. The density of the groundwater samples was about one sample per 2.5 square km.

Groundwater is the most important source of drinking water for most of the population of the Slovak Republic, covering approximately 90% of inhabitants [34]. Approximately 10% of the Slovak population uses water from individual wells for drinking and cooking purposes. About 50% of the population is supplied with drinking water from local water companies using local water resources with a low discharge (less than 10 L·s^−1^), which is captured and distributed to water supply pipes in the vicinity of villages. Only in southern Slovakia (especially in the Quaternary sediments) is the population supplied from large water resources that are distributed across distances of 50–100 km. The rate of consumed bottled drinking water, mineral water, and various non-alcoholic drinks is currently at about 20% of daily consumption [35]. In this study, we consider groundwater and drinking water as one. We are aware of some inaccuracies, which may limit our results, but the magnitude of the dataset (more than 20,000 chemical analyses and of more than 30 chemicals) and the effectiveness of the mathematical methods of data analysis used (artificial neural networks) significantly reduce those uncertainties.

We transformed the EI data on water chemical composition into a form compatible with the data on health indicators to give one value for each administrative-territorial unit of the Slovak Republic at the municipal level (2883 municipalities). Based on the analytical data we have compiled, the surface distribution of each evaluated chemical element or compound is shown in the form of pixel maps (1 pixel represents surface 1 square km), using MapInfo Professional 9.0 software (Pitney Bowes Inc., Stamford, CT, USA). An average elemental concentration for each pixel was computed through the common inverse distance interpolation gridding method based on averaging 10 samples that are the nearest to the pixel center. The average value for chemicals for specific administration units (villages, districts, and the entire Slovak Republic) was then calculated as the mean value of each pixel falling under the administration units. They are available in both table and map forms on the website of the project Geohealth [36].

The EI set of evaluated chemicals in the groundwater with respective mean values for the Slovak Republic is reviewed in Table 1 [33].

### 2.2. Health Indicators

The health of the Slovak population, which is approximately 5.5 million inhabitants, was evaluated based on health indicators (HI), which are indicators of the demographic growth and health of inhabitants. An HI is a variable that expresses the health of inhabitants in a society through direct measurement or observation [37]. For the evaluation of the impact of chemical composition of groundwater on the health of the population, we used the dataset of 14 HI, reviewed in Table 2 together with a description of the methods used for their calculations. For data evaluation, we have selected basic demographic indicators, namely life expectancy at birth and potential years of lost life for all causes of death. Additionally, we evaluated the four most significant causes of death in Slovakia that can be potentially associated with the environment as an influencing factor; these are cardiovascular diseases (CVD), oncological diseases (OD), diseases of the gastrointestinal tract (DGT), and diseases of the respiratory system (DRS). HI are expressed in the form of relative or indirect age-standardized mortality for selected diagnoses in accordance with the international classification of diseases (ICD, 10th revision [38]).

**Figure 1:**
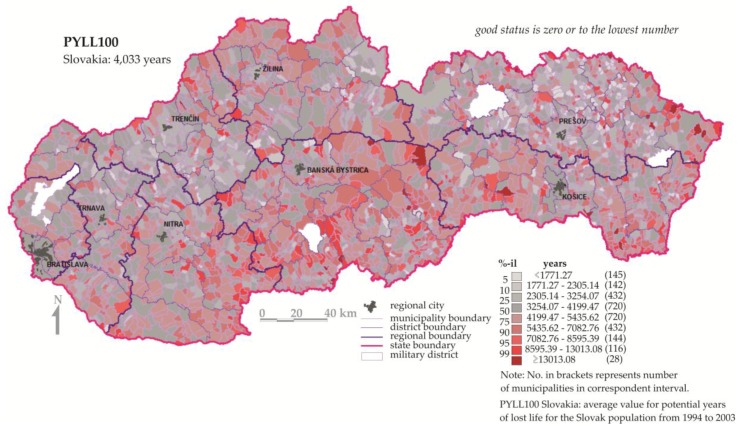
Potential years of lost life in the Slovak Republic at levels of municipalities.

The data source was the database of the Statistical Office of the Slovak Republic [39]. All HI were calculated as a cumulative function for each territorial unit assessed over a ten-year period, spanning from 1994 to 2003. The calculation methodology and standardization of HI was carried out according to recommendations made by the WHO and previous works [37,40,41,42,43]. Our data thus represents average values for a 10-year period for each of the 2883 Slovak municipalities. Map visualization of HI is presented for potential years of lost life (PYLL100) in Figure 1. Other evaluated health indicators are available on the website of the project Geohealth [36].

The linking of environmental and health data was performed using standard correlation methods, as well as artificial neural networks (ANN). We also compared the health of residents according to variable geological structures, and thus in relation to various chemical compositions of groundwater.

### 2.3. Statistical Analysis

The Statistical analysis of the relationship between EI and HI was based on standard methods of data correlation, including a linear (Pearson) correlation, a coefficient, and a non-parametric (Spearman) order correlation coefficient. Statistical significance of calculated correlation coefficients was evaluated based on the level of significance *p* as follows: *p* < 0.05—verified dependence (+), *p* < 0.01—high dependence (++), *p* < 0.001—very high dependence (+++).

### 2.4. Neural Network Analysis

Investigation of the relationships between two different variables is the domain of statistics. However, the selection of appropriate methods of linking two databases is required to measure relevant interdependency and relationships. Correlation coefficients express the intensity of stochastic dependence between two variables, demonstrating dependent relationships between measured attributes. Classical Pearson correlation coefficients express the degree of simple linear dependence of two variables. Spearman correlation coefficients are a measure of monotonic dependence. Our data was not normally distributed, but unevenly distributed, and often spoiled by errors, being incomplete, and/or exhibiting high variability. Data have all the attributes of common life. When a doctor reported more incidences of a particular cause of death, a registrar responsible for completing the statistical reports selected only one diagnosis—especially the first listed diagnosis (e.g., the most common are cardiovascular diseases or renal failure). However, the selected diagnosis may not always be the main or the most fundamental cause of death (e.g., in case of an oncological patient who was treated with chemotherapeutic agents, which is usually not ranked in the first place on the list of diagnoses). Classical methods of regression analysis may not explore the situation fully in its complexity and may lead to wrong conclusions. Complex situations merit complex analytical approaches [44]. Therefore, for the analysis of relationships between chemical composition of groundwater and particular HI we used ANN. The order of effects of the chemicals in groundwater on HI was determined from the value of the sensitivity coefficient *s_r_*. HI are influenced by those chemical elements for which the sensitivity coefficient is greater than one. In order to identify the influential chemicals from the point of view of the groundwater chemical composition, 200 networks were calculated. Selecting 200 networks has proven to be fully satisfactory, because for the next networks the value of correlation coefficient does not increase, but stagnates or declines. Despite the satisfactory performance (reliability) of the network, the impact of various EI was relatively low and was different for each created network. Therefore, the most influential chemicals were ordered based on median values of the *s_r_* of 50 of the best networks with the highest correlation coefficient. This approach has been used by several authors [45,46,47].

Based on the median values of *s_r_* (from the best 50 networks) calculated for each chemical element, we assess the influence of the chemical element on particular HI. The influence increases with the increase of the *s_r_* value. Chemical elements with an *s_r_* < 1 are defined as not influential on HI. Statistical significance of the calculated coefficients *s_r_* is characterized by the coefficient of determination R^2^. The statistical significance of *s_r_* increases with the increase of R^2^.

The results of calculations of ANN necessary for the determination of the shape of dependence between EI and HI were verified through the method of deciles. The range of concentrations of each element evaluated in groundwater was divided into deciles. In the next step, we found the centroid of the points where the *x*- coordinate belonged in individual deciles. Subsequently we found the second-degree polynomial that ran a straight line through the center of deciles 2 to 9. The conformity for the very influential elements was excellent. In the decline of the element influence the consensus exists, but the similarity decreases [44].

**Figure 2:**
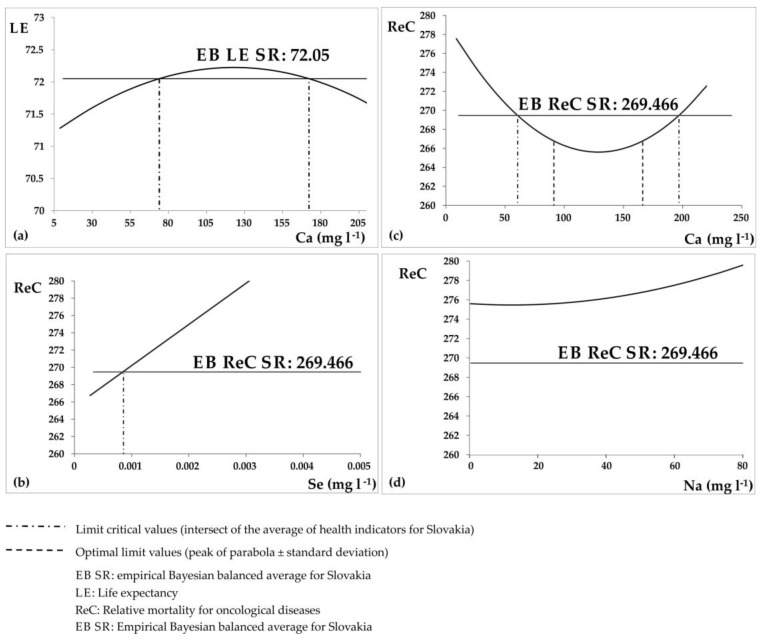
Derivation of limit values for environmental indicators in relation to their influence on health indicators. (**a**) relationship between life expectancy and calcium groundwater contents; (**b**) relationship between relative mortality for malignant neoplasms and selenium groundwater contents; (**c**) relative mortality for malignant neoplasms and calcium groundwater contents; (**d**) relative mortality for malignant neoplasms and sodium groundwater contents.

The ANN method is very useful also for derivation of limit values for chemical elements in groundwater in relation to particular HI in that it can derive content levels for evaluated chemicals in which HI have the most favorable values. We define two types of limit values: limit (critical) values and optimal limit values. Limit (critical) contents represent the intersection of the model curve of chemical content with the average value of HI. Optimal limit contents represent, in the case of a curve shape (convex parabola) intersection of the peak with an average value of the HI ± standard deviation of the HI. In the case that the model curve of the chemical content does not intersect the average value of the HI, we were not able to determine the limit values. For many chemical elements, the limit value (upper or lower limit) does not exist. This means that increasing or decreasing the content of chemical elements in groundwater does not have any influence on the health of the population. The derivation of limit values for EI is shown in Figure 2. We used the empirical Bayesian balanced average as the average value of HI instead of real values. The advantage of this approach is that it takes into account the number of inhabitants in single municipalities [30,48,49].

### 2.5. Comparison of Health According to Geological Structure

The geological structure of the Slovak Republic is rather complicated. It is characterized by alteration of rocks with various geneses, ages, and therefore various mineralogical and petrographic characteristics and variable geochemical backgrounds [50]. It is reflected in the variable chemical composition of groundwater, which can have various influences on the health of the population.

The geological structure of the Slovak Republic has been divided into eight main units, which are ordered according to their influence on the health of Slovak residents (from the most favorable to the most unfavorable) [33] as follows:

1. Paleogene Flysch: mainly sandstones, shales, and claystones
2. Carbonate-Silicate Mesozoic and Paleogene: mainly marl, marly limestones, dolomites, sandstones, and shales
3. Carbonate Mesozoic and Basal Paleogene: mainly limestones, dolomites, and carboniferous conglomerates
4. Neogene: mainly clays, claystones, conglomerates, sands, and gravels
5. Quaternary: mainly gravel, sand, clay, and rock fragments
6. Crystalline: mostly granites, gneisses, and migmatites
7. Paleozoic: mostly metasediments, and metavolcanics
8. Neovolcanic rocks: mainly andesites, basalts, and their volcanoclastics

Generally, the most favorable rock environments for human health were defined as carbonatic rocks and the most unfavorable as silicate rocks.

Subsequently, we divided our data by chemical composition of groundwater for both EI and HI. HI were put into partial datasets according to the geological units, in which they are compared analyzed through the aforementioned statistical methods in Section 2.3 and Section 2.4.

## 3. Results

Selected results of linear and Spearman correlations among EI and HI for geological environment as a whole are summarized in Table 3. All results are available on the Geohealth website [36].

In Table 4 and Table 5 we summarize the results of the calculations from the ANN for the geological environment as a whole. Table 4 provides a review of the results of sensitivity coefficients for the most influential chemical elements and parameters in the groundwater and evaluated the HI together with the order of influence for single elements. In Table 5 the results of calculations of ANN for life expectancy (LE) are shown including derived limit values for the most influential chemical elements and parameters in the groundwater. All results of ANN calculations for evaluated HI are published on the Geohealth website [36].

The mean values of evaluated HI for the most favorable geological units (Paleogene Flysch—1) and the most unfavorable geological units (Neovolcanic rocks—8) in relation to HI are reviewed in Table 6 together with the mean values for the Slovak Republic as well as two selected districts of the SR (Krupina and Bardejov). The Krupina district is entirely built on the rock environment of Neovolcanics (in terms of HI, it is the least favorable geological environment) and the resident population is supplied only by drinking water from local groundwater sources of this district. The Bardejov district is entirely built by Flysch Paleogene rocks (in terms of HI, it is the most favorable geological environment) and the resident population is supplied from local groundwater sources of this district. The mean contents of 10 of the most influential chemical elements and parameters on HI according to ANN, together with two typical potentially toxic elements (PTEs) arsenic and lead, are shown in the aforementioned units in Table 7. Mean values of EI and HI for individual geological units are available on the Geohealth website [36].

## 4. Discussion

### 4.1. Statistical Analysis

Our variables EI and HI do not have normal distribution; analysis reveals that dependences are generally not linear, and are often even monotonous. That is why we do not find the achieved results reliable. Correlation coefficients are very low in both correlations, and in more than 90% of cases they range is characterized as ±<0.1. However, a very important fact is that the correlation coefficients for Ca, Mg, water hardness (Ca + Mg), and evaluated HI (with the exception of life expectancy) in almost all cases show negative values dominantly, and at levels that are statistically very high in significance. This is also true in the cases of other HI not reviewed in Table 3. This fact indicates deterioration in values of all evaluated HI for mortality at low levels of Ca and Mg contents, and for water hardness of the groundwater in the Slovak Republic. The reverse situation is documented for the HI of life expectancy, for which we observe positive values of correlation coefficients in the case of Spearman, as well as linear correlation, at levels that are statistically very high in significance. This clearly indicates a trend of increased levels of Ca and Mg contents and water hardness extends human life (i.e., life expectancy is higher).

### 4.2. Neural Networks

We find the results obtained through ANN calculations more representative (compared to linear and Spearman correlations) because they eliminate the inhomogeneity of the datasets. Based on the obtained results we documented, Ca + Mg, Ca, Mg, TDS, HCO_3,_ and SO_4_ as the most influential elements and parameters of chemical composition of groundwater for individual HI (Table 4 and Table 5). These six parameters are found in the group of the first 10 most influential EI in all evaluated HI. Other EI − Cl, NO_3_, SiO_2_, Na, and K were ranked among the 10 most influential parameters only in the case of some evaluated HI. Their averaged influence (xP) on HI is relatively low (7.85–16) and their mean levels of sensitivity coefficient are low (*s_r_* < 1.01). Three groups of chemical elements and parameters among the most influential EI on HI can be clearly identified. The first group contains Ca, Mg, and Ca + Mg. We attribute the highest influence on HI to these three EI. They show the highest levels of *s_r_*. The second group of EI (TDS and HCO_3_) is considered to have only a statistical relationship with HI (i.e., the second group has only a statistical influence on HI). This is demonstrated by the fact that the chemical composition of groundwater in the Slovak Republic is mainly of a Ca-Mg-HCO_3_ character. TDS and HCO_3_ can be generally seen as indicators of Ca and Mg groundwater content. HCO_3_ is the most common anion in groundwater in the Slovak Republic, and its concentrations are mainly due to Ca and Mg cations (mineralization of water that occurs due to the dissolution of carbonates). Similarly, values of groundwater mineralization (TDS) depend mainly on the Ca and Mg contents (the most common cations) and HCO_3_ (the most common anion) content in the groundwater of the Slovak Republic [32]. The third group of influential elements consists of SO_4_, Cl, and NO_3_. These three parameters are typical of anthropogenic groundwater contamination in the Slovak Republic. Their influence is, based on levels of sensitivity coefficients *s_r_*, markedly lower compared to the influence of Ca, Mg, and Ca + Mg. The important fact regarding these three parameters is that their increased contents in the groundwater of the Slovak Republic is due to anthropogenic contamination, and are accompanied mainly by increased contents of Ca and Mg, which were documented in this study as the most influential parameters in relation to HI. Therefore, the mentioned anions do not have significant influence on the health of the Slovak population. This statement does not deny any potential negative effects of nitrates, chlorides, and sulphates on human health at all. All of these chemicals can have significantly adverse health effects at contents locally increased in particular groundwater sources. Such highly contaminated groundwater sources are not used for drinking purposes and therefore we do not consider the influence of these three parameters in groundwater as a significant determinant on the health of the population within the Slovak Republic. A very important fact is that all potentially toxic elements, such as As, Pb, Hg, Zn, Sb, and others have a very low influence on HI, or are characterized as not influential on HI. In the majority of cases, their sensitivity coefficients are lower than, 1 or they are very low (*s_r_* < 1.01). This finding is fully aligned with our current knowledge on the low impact of potentially toxic elements on the health of the population in contaminated abandoned mining areas present in the Slovak Republic [51].

Based on the results of ANN calculations, Ca, Mg and water hardness (Ca + Mg) were clearly identified as the most influential EI for evaluated HI. Other evaluated EI are found to be less influential, or to have a stochastic relationship to HI. Therefore, we will not discuss them further.

Ca and Mg are important intracellular cations, which are significantly involved in many enzymatic systems. They are essential for several biological processes, including hematopoiesis, the proper functioning of the heart, and in the prevention of oncological diseases [9,52]. The significance of both elements in drinking water for cardiovascular diseases has been documented many times in academic literature, mainly for the association of Ca and Mg deficits and increased incidence or mortality rates for CVD [21,22,23,24]. However, there are very few works linking the incidence or mortality rates of OD with Ca and Mg deficiency in drinking water [25,26,27,28,29,30,53]. Nriagu et al. link increased carcinogenity of desalinated water with electrolyte disturbance and hypomagnesemia [54]. Generally, the epidemiologic studies show that the influence of Ca and Mg on the increased occurrence of oncological diseases is ambiguous. Some of the studies attribute an increased incidence of these diseases (cancers of the breast, prostate, stomach, and digestive tract) to raised Ca or Mg contents in human tissues and fluids, while some report exactly the opposite results [55,56,57,58,59]. However, these studies did not deal with water intake, but with artificial supplementation of calcium consumed from diet and supplements.

We were not able to find any reference in any academic literature worldwide dealing with an increased incidence or mortality rate for diseases of the gastrointestinal or respiratory systems and deficits of Ca and Mg in drinking water. Only one Russian ecological study describes a significantly higher incidence of stomach and duodenal ulcer related to soft water with water hardness less than 1.5 mmol·L^−1^ (Lutai, 1992 in Kožíšek, 2003 [60]). In our study, observations are of the highest differences in mortality for only diseases of the gastrointestinal and respiratory systems.

Silicate geological environment with low mineralized groundwater/drinking water is generally less favorable in relation to the increased mortality from DGT and DRS compared to carbonate geological environment associated with higher Ca and Mg groundwater contents. Currently, we are not able to find clear and relevant explanation. It is definitely recommended that more specific studies should be done in the future in other countries to confirm or confute this finding.

Mortality for CD, OD, DGT, and DRA represent about 80%–85% of the causes of death in Slovakia [61]. Increased mortality of Slovak population for these diseases is documented in silicate geological environment at national as well as regional level (the Krupina district). It is strongly reflected in demographic indicators represented in this study by life expectancy (LE) and potential years of lost life (PYLL100). This difference can most markedly be seen in the comparison of the two discussed districts. The difference in life expectancy is more than four years, and in potential years of lost life the difference is more than 100% to the detriment of the Krupina district. It is therefore evident that the deficit of Ca and Mg or water hardness are significantly reflected in all the main causes of death in Slovakia, including cardiovascular and oncological diseases, as well as diseases of the gastrointestinal and respiratory systems. Moreover, with increased concentrations of these chemical elements, life expectancy is higher (i.e., life expectancy is higher).

### 4.3. Comparison of Health Regarding Geological Structure

Based on the comparison of HI in single geological units (Table 6), significant differences in life expectancy, potential years of lost life, and mortality rates are documented. Carbonate geological units (Paleogene Flysch—1) are characterized with significantly more favorable levels of practically all HI compared to silicate geological units (Neovolcanic rocks—8). For example, life expectancy in Paleogene Flysch areas is more than 2.5 years higher (LE = 73.69 years) than in areas with a geological unit of Neovolcanic rocks (LE = 71.11 years). A similar situation can be observed in the case of potential years of lost life (PYLL100). Its level is about 20% lower in areas with Paleogene Flysch rocks (3874.38 years), meaning there are more favorable environmental conditions than in Neovolcanic rocks (4586.18 years). The differences in relative and standardized mortality for cardiovascular and oncological diseases and diseases of gastrointestinal and respiratory systems between these two geological units range from 20% to 100% to the detriment of silicate geological units. Even higher differences in the levels of HI are observed when comparing the two districts, namely the Bardejov district (the most favorable geological environment—Paleogene Flysch rocks) and the Krupina district (the most unfavorable geological environment—Neovolcanic rocks). The following are the differences to the detriment of the Krupina district: lower life expectancy by more than four years, PYLL100 (more than 90% worse), and relative mortality for diseases of the gastrointestinal and respiratory systems more than three times higher than in the Bardejov district. The situation in the case of other HI is similar. More significant differences in the levels of HI in the two evaluated districts compared to differences documented between single geological units can be attributed to the fact that silicate rock environment is less aquiferous. The resident population in this area is often supplied by drinking water from more distant, dominantly carbonate units (i.e., with markedly higher Ca and Mg groundwater contents), which have generally much greater water-bearing capacity. Therefore, we attribute these differences in health indicators documented between carbonate and silicate geological units and both districts mainly to different levels of Ca Mg contents and water hardness.

The contents of these three chemical elements and parameters in groundwater are significantly higher in carbonate geological units than in silicate geological units (Table 7). We did not observe significant differences between the contents of other chemicals within the evaluated geological units.

### 4.4. Proposed Limit Values

The most important output of our work is the definition of limit values for evaluated EI, for which we document the lowest mortality rates or for maximum life expectancy. We review here the limit values for evaluated HI together with recommended (not obligatory) values defined by the Slovak guidelines for drinking water for comparison in Table 8. If we have to take into account the importance of a particular HI, we will characterize life expectancy and potential years of lost life as the most significant. They reflect all other HI. Moreover, these are followed by other significant HI, such as the mortality rate for cardiovascular diseases (about 48% of all causes of death) and that of oncological diseases (about 25% of all causes of deaths). The mortality rate for diseases of the gastrointestinal system (about 6% of all causes of deaths) and that of the respiratory system (about 5% of all causes of death) have a lower level of significance. We cannot define the levels only on a mathematical basis. For about half of the HI, the limit value does not exist or cannot be defined (Table 8). The absence of limit values means that increasing or decreasing content of chemical elements does not have an influence on the HI. In defining limit values, we also have to take into account the potential adverse health effects of very hard water. One of the potential health effects of hard water that should be mentioned is the formation of urinary stones. However, some epidemiological studies did not confirm this relationship [62,63]. Currently there is no direct evidence that increased water hardness can cause adverse health effects [60], except for extremely high Mg water content (hundreds of mg·L^−1^) that cause diarrheal diseases. Among the other adverse effects of hard water are sensory properties, such as unfavorable tastes, formation of coatings on the surface of coffee or tea glasses, and the loss of aromatic substances from food and beverages caused by binding with Ca carbonate. From the technological point of view even hard water is not favorable because of scale formation, but it is also the case that soft water has corrosive abilities.

Optimal water hardness from the point of view of human health is hard to determine. Most authors recommend the most favorable values for Mg at a minimum of 20–30 mg·L^−1^, for Ca 40–80 mg·L^−1^ and for water hardness 2–4 mmol·L^−1^ [9].

After taking into account our calculations and all other known facts we propose the following limit values for Ca, Mg, and water hardness used for public supply in the Slovak Republic. For water used for drinking we propose values of Ca > 50 mg·L^−1^, Mg > 25 mg·L^−1^, and water hardness > 2 mmol·L^−1^ (Table 9). In the case of Ca, we propose a lower limit value compared to that derived by ANN calculations in this study, due to the generally reviewed fact that the potential health effects are more likely attributed to Mg in drinking water than to Ca [64]. Both elements almost always occur together in groundwater in the Slovak Republic, mainly in the Ca/Mg ratio 3:1 (calculated from mg·L^−1^) in the case of both low mineralized and high mineralized water. Therefore, we are not able to evaluate the health effects of Ca and Mg separately. The proposed limit values for Ca, Mg and water hardness are about two times higher compared to the recommended values defined in the Slovak guideline for drinking water (Table 8). Following this fact, we recommend increasing existing limits of these parameters to reach a lower level of mortality for the most common causes of death and a longer lifetime.

In Slovakia, about 25% of groundwater sources are characterized by lower Ca, Mg, and Ca + Mg concentration levels than the proposed limit values.

Our proposed lower limit values are aligned with recommended limit values reported by other authors [9]. However, levels of water hardness that are too high could have unfavorable effects on consumers. Water with increased hardness (>5 mmol·L^−1^) or with increased Ca contents (>180 mg·L^−1^) and Mg contents (>50 mg·L^−1^) do not naturally occur in the territory of the Slovak Republic and they are not used for drinking purposes. Therefore, we do not find it relevant to define upper limit values.

### 4.5. Other Impacts Besides Environmental Factors

In conclusion, we mention some other factors that can be characterized as confounding factors for our results. In addition to the chemical composition of the groundwater already discussed, the health of inhabitants depends on a series of other factors, such as eating habits, lifestyle, quality and access to health care, air pollution, socio-economic conditions, etc. Such data is not available for particular Slovak municipalities but is available in other selected areas and districts.

Therefore, below we provide a review of available information concerning the Krupina district (with the most unfavorable geological environment) and the Bardejov district (with the most favorable geological environment), where we document the highest differences in the levels of mortality for the discussed causes of death, as well as that of life expectancy.

Both Krupina and Bardejov represent typical rural districts situated in mountain areas of the Slovak Republic. The population in both districts lives mainly in family houses. Most residents grow vegetables and fruit in their gardens for their own consumption. Regarding air quality, we can state that neither in the two districts, nor in their surroundings, is there any significant source of air pollution (e.g., industrial chemicals, coal power plant). The level of air pollution in both districts is low and that is why no local station for monitoring of air quality is situated there [65]. We also document very low levels of soil contamination in both evaluated districts [66,67].

Probably a very important confounding factor is rate of Gypsy population. The Gypsy population is characterized by a significantly worse socio-economic level; it has a lower health status and also lower life expectancy in comparison with other Slovak population. In the Bardejov district with documented more favorable health status of resident population a total number of inhabitants of Gypsy population is approximately two times higher than that in the Krupina district (Table 10).

Other important socio-economic factors that could have some impact on HI include registered levels of unemployment, as well as the average salary of local residents. Both factors show more unfavorable levels in the Bardejov district compared to the Krupina district.

There are other health determinants regarding lifestyle and health-care that influence human health both positively and negatively, and the available data on them are reviewed in Table 10. Based on the comparison of the reviewed data, we can conclude that there are no significant differences between the listed health determinants in either of the discussed districts. On the other hand, slightly but not significantly better values for these factors can be observed in the Krupina district, where significantly worse health and shorter life expectancy were reported.

## 5. Conclusions

In our study, we assessed the health of the population in relation to the chemical composition of groundwater across the entire Slovak territory (consisting of about 5.5 million inhabitants, 50,000 square kilometers). Environmental and health data were evaluated for all 2883 Slovak municipalities. Data evaluation through three various methods (Spearman and linear, artificial neural networks, and the comparison of the health of the population living in various geological environments with different Ca and Mg groundwater levels) revealed that Ca, Mg, and water hardness are the most influential parameters of the chemical composition of groundwater on the health of the population of the Slovak Republic.

Based on the achieved results we can conclude that the health of the population in the Slovak population, together with their life expectancy, is significantly influenced by the contents of Ca, Mg, and water hardness in the groundwater. Mortality mainly for cardiovascular and oncological diseases, as well as diseases of the gastrointestinal and respiratory systems, is markedly lower at concentration ranges of these parameters in groundwater as follows: Ca 78–155 mg·L^−1^, Mg 28–54 mg·L^−1^, and water hardness 2.9–6.1 mmol·L^−1^. Worse health status and lower life expectancy are observed at low, deficit contents of these parameters in groundwater. Our derived limit values for these three parameters are about 2 times higher compared to limits defined within the Slovak guideline for drinking water. We propose to increase them in the case of drinking water used from public supply at the following concentrations: Ca > 50 mg·L^−1^, Mg > 25 mg·L^−1^, and Ca + Mg > 2 mmol·L^−1^. The increase of Ca and Mg contents may be reached by proper water treatment based on the process of water re-carbonization. Based on this increase, gradual and continual improvement of the health of the population can be expected. Based on the achieved results, we recommend that the World Health Organization consider revising their definition of drinking water quality standards for Ca and Mg contents and water hardness.

## Figures and Tables

**Table 1:** Characteristics of chemical composition of groundwater in the Slovak Republic (mean values).

Groundwater (*n* = 20,339)												
pH	TDS	COD_Mn_	Ca + Mg	Li	Na	K	Ca	Mg	Sr	Fe	Mn	NH_4_
7.33	629.75	2.18	3.5	0.019	20.34	11.10	93.56	28.29	0.36	0.17	0.12	0.10
F	Cl	SO_4_	NO_2_	NO_3_	PO_4_	HCO_3_	SiO_2_	Cr	Cu	Zn	As	Cd
0.13	32.96	79.32	0.11	38.76	0.20	303.85	18.21	0.0013	0.0026	0.2673	0.0019	0.0010
Se	Pb	Hg	Ba	Al	Sb	^222^Rn	^226^Ra					
0.0010	0.0014	0.0001	0.0747	0.0297	0.0009	14.46	0.053					

Note: TDS—total dissolved solids; COD_Mn_—chemical oxygen demand with oxidizing agent potassium permanganate, data except of pH in mg·L^−1^, Ca + Mg in mmol·L^−1^, ^222^Rn and ^226^Ra in Bq·L^−1^.

**Table 2:** Evaluated health indicators of the Slovak Republic.

Health Indicator	Description of Indicator	Method of Calculation	Units	Mean SR *
Demographic indicators describing age structure of municipalities				
LE	life expectancy at birth—population	cumulative calculation of all years of life during lifetime/No. of living persons at the beginning of the year	years	72.60
Premature mortality				
PYLL100	potential years of lost life	100,000 × (the sum of the years of people up to the age of nearly 65 years (deaths at age between 1 and 64 years)/number of inhabitants)	years	4033.00
Relative mortality for selected cause of death				
ReC00-C97	malignant neoplasms	100,000 × (No. of deaths for selected cause/number of inhabitants)	No. of deaths per 100,000 inhabitants	212.79
ReI00-I99	diseases of the circulatory system			531.05
ReJ00-J99	diseases of respiratory system			58.08
ReK00-K93	diseases of the digestive system			45.83
Standardized mortality for selected cause of death				
SMRC00-C97	malignant neoplasms	indirect age-standardized mortality rate of inhabitants to the Slovak standard (19 age groups)	%	100
SMRI00-I99	diseases of the circulatory system			100
SMRJ00-J99	diseases of respiratory system			100
SMRK00-K93	diseases of the digestive system			100
Potential years of lost life for selected cause of death				
PYLLC00-C97	malignant neoplasms	100,000 × (the sum of the years of people up to the age of nearly 65 years (deaths at age between 1 and 64 years)/number of inhabitants)	years	1005.20
PYLLI00-I99	diseases of the circulatory system			866.19
PYLLJ00-J99	diseases of respiratory system			172.69
PYLLK00-K93	diseases of the digestive system			334.80

Note: Health indicators are classified according to International classification of diseases (ICD), 10th revision [38], * SR—mean for the Slovak Republic for the period 1994–2003.

**Table 3:** Pearson and Spearman correlation between environmental indicators (EI) and health indicators (HI) for the geological environment as a whole.

Parameter	Linear Correlation			Spearman Correlation		
	*r*	*p*	Significance	*R*	*p*	Significance
Ca + Mg and LE	0.140	0.000	+++	0.181	0.000	+++
NO_3_ and LE	−0.021	0.392	−	0.069	0.005	++
As and LE	0.020	0.411	−	−0.078	0.001	++
Ca + Mg and PYLL100	−0.130	0.000	+++	−0.187	0.000	+++
NO_3_ and PYLL100	−0.001	0.960	−	−0.077	0.002	++
As and PYLL100	−0.017	0.484	−	0.083	0.001	+++
Ca + Mg and ReC00-C97	−0.085	0.000	+++	−0.134	0.000	+++
NO_3_ and ReC00-C97	−0.050	0.043	+	−0.112	0.000	+++
As and ReC00-C97	−0.001	0.960	−	0.080	0.001	++
Ca + Mg and SMRC00-C97	−0.033	0.175	−	−0.038	0.119	−
NO_3_ and SMRC00-C97	0.012	0.618	−	−0.004	0.861	−
As and SMRC00-C97	0.006	0.798	−	0.086	0.000	+++
Ca + Mg and PYLLC00-C97	−0.079	0.001	++	−0.095	0.000	+++
NO_3_ and PYLLC00-C97	−0.028	0.258	−	−0.042	0.086	−
As and PYLLC00-C97	−0.001	0.971	−	0.106	0.000	+++
Ca + Mg and ReI00-I99	−0.083	0.001	+++	−0.151	0.000	+++
NO_3_ and ReI00-I99	−0.031	0.198	−	−0.092	0.000	+++
As and ReI00-I99	−0.013	0.586	−	0.030	0.224	−
Ca + Mg and SMRI00-I99	−0.023	0.351	−	−0.046	0.061	−
NO_3_ and SMRI00-I99	0.077	0.002	++	0.052	0.034	+
As and SMRI00-I99	−0.014	0.578	−	0.039	0.112	−
Ca + Mg and PYLLI00-I99	−0.084	0.001	+++	−0.121	0.000	+++
NO_3_ and PYLLI00-I99	0.042	0.083	−	0.002	0.929	−
As and PYLLI00-I99	−0.020	0.421	−	0.091	0.000	+++
Ca + Mg and ReJ00-J99	−0.108	0.000	+++	−0.138	0.000	+++
NO_3_ and ReJ00-J99	−0.057	0.020	+	−0.111	0.000	+++
As and ReJ00-J99	−0.003	0.912	−	0.090	0.000	+++
Ca + Mg and SMRJ00-J99	−0.066	0.007	++	−0.084	0.001	+++
NO_3_ and SMRJ00-J99	−0.010	0.693	−	−0.056	0.023	+
As and SMRJ00-J99	0.004	0.871	−	0.081	0.001	+++
Ca + Mg and PYLLJ00-J99	−0.025	0.302	−	−0.079	0.001	++
NO_3_ and PYLLJ00-J99	0.009	0.715	−	0.004	0.856	−
As and PYLLJ00-J99	−0.006	0.806	−	0.058	0.018	+
Ca + Mg and ReK00-K93	−0.049	0.047	+	−0.119	0.000	+++
NO_3_ and ReK00-K93	0.075	0.002	++	−0.038	0.116	−
As and ReK00-K93	0.001	0.959	−	0.171	0.000	+++
Ca + Mg and SMRK00-K93	−0.039	0.112	−	−0.088	0.000	+++
NO_3_ and SMRK00-K93	0.105	0.000	+++	0.007	0.780	−
As and SMRK00-K93	0.018	0.456	−	0.168	0.000	+++
Ca + Mg and PYLLK00-K93	−0.041	0.092	−	−0.079	0.001	++
NO_3_ and PYLLK00-K93	0.079	0.001	++	0.006	0.800	−
As and PYLLK00-K93	0.028	0.248	−	0.156	0.000	+++

Note: *r*—Pearson correlation coefficient, *R*—Spearman correlation coefficient, *p*—value; level of significance < 0.05 = not verified dependence, level of significance *p* = 0.05—verified dependence (+), *p* = 0.01—high dependence (++), *p* = 0.001—very high dependence (+++).

**Table 4:** Coefficients of sensitivity and order of influence for 10 of the most influential elements and parameters in groundwater in relation to HI according to calculations through artificial neural network (ANN).

Parameter	1		2		3		4		5		6		7		8		9		10		11		12		13		14		xP
	*s_r_*	P	*s_r_*	P	*s_r_*	P	*s_r_*	P	*s_r_*	P	*s_r_*	P	*s_r_*	P	*s_r_*	P	*s_r_*	P	*s_r_*	P	*s_r_*	P	*s_r_*	P	*s_r_*	P	*s_r_*	P	
Ca + Mg	1.419	1	1.115	1	1.027	3	1.370	1	1.590	1	1.057	1	1.003	3	1.677	1	1.001	6	1.180	1	1.044	1	1.046	1	1.003	4	1.169	1	1.92
Mg	1.153	3	1.027	3	1.005	8	1.150	3	1.255	3	1.009	7	1.004	1	1.291	3	1.002	4	1.065	3	1.004	4	1.002	6	1.004	3	1.063	3	3.92
Ca	1.246	2	1.048	2	1.013	4	1.211	2	1.346	2	1.015	5	1.003	2	1.387	2	1.003	3	1.108	2	1.008	3	1.006	3	1.004	2	1.100	2	2.62
TDS	1.086	4	1.003	5	1.074	1	1.053	4	1.008	4	1.015	6	1.001	8	1.018	4	1.016	1	1.051	4	1.016	2	1.002	7	1.010	1	1.028	4	3.92
HCO_3_	1.012	8	1.013	4	1.034	2	1.026	5	1.005	5	1.023	4	1.002	4	1.006	5	1.005	2	1.028	5	1.002	5	1.010	2	1.003	6	1.012	5	4.38
SO_4_	1.004	9	1.002	7	1.0094	5	1.009	7	1.001	8	1.006	8	1.001	10	1.001	10	1.001	5	1.006	8	1.001	10	1.003	5	1.001	7	1.003	9	7.77
Cl	1.003	11	1.002	9	1.007	6	1.027	6	1.001	9	1.029	2	1.001	5	1.001	11	1.000	13	1.021	6	1.002	6	1.002	8	1.001	8	1.003	8	7.85
NO_3_	1.003	10	1.001	11	1.006	7	1.004	8	1.001	10	1.003	9	1.001	11	1.002	6	1.001	8	1.004	9	1.001	8	1.001	11	1.001	11	1.001	10	9.31
SiO_2_	1.002	13	1.002	8	1.001	12	1.003	10	1.000	17	1.027	3	1.001	6	1.001	14	1.000	11	1.014	7	1.000	13	1.001	9	1.000	21	1.008	6	10.77
Na	1.0434	7	1.001	12	1.003	9	1.002	9	1.000	16	1.002	12	1.001	12	1.001	13	1.000	14	1.001	13	1.001	7	1.003	4	1.001	9	1.000	19	11.31
K	1.0732	6	1.000	15	1.000	17	1.001	12	1.000	20	1.000	17	1.001	20	1.001	15	1.000	16	1.000	17	1.000	14	1.001	10	1.000	13	1.000	28	16.00

Note: *s_r_*—coefficient of sensitivity; P—order of influence; xP—arithmetic mean of order of influence for all evaluated HI; 1—LE; 2—PYLL100; 3—ReC00-C97; 4—ReI00-I99; 5—ReJ00-J99; 6—ReK00-K93; 7—SMRC00-C97; 8—SMRI00-I99; 9—SMRJ00-J99; 10—SMRK00-K93; 11—PYLLC00-C97; 12—PYLLI00-I99; 13—PYLLJ00-J99; 14—PYLLK00-K93.

**Table 5:** Results of calculations of ANN and derived limit values for 10 the most influential chemical elements and parameters in groundwater of the Slovak Republic in relation to life expectancy (LE).

Order	Parameter	*s_r_*	R^2^	Limit Content		Optimal Content		Evaluated Function of Dependence	Contents *	
				LL	UL	LL	UL		Min	Max
1	Ca + Mg	1.419	0.997	2.98	does not exist	not defined	does not exist	concave parabola	0.35	7.97
2	Ca	1.246	0.975	73.95	172.21	not defined	not defined	concave parabola	9.83	201.01
3	Mg	1.152	0.975	18.13	does not exist	not defined	does not exist	concave parabola	2.45	97.75
4	TDS	1.086	0.899	358.46	does not exist	does not exist	does not exist	concave parabola	87.30	1412.30
5	COD_Mn_	1.081	0.994	does not exist	2.27	does not exist	does not exist	concave parabola	0.75	7.48
6	K	1.073	0.964	does not exist	9.85	does not exist	does not exist	concave parabola	0.27	153.15
7	Na	1.043	0.977	does not exist	24.07	does not exist	does not exist	concave parabola	0.71	119.69
8	HCO_3_	1.012	0.993	250.79	does not exist	does not exist	does not exist	concave parabola	16.57	592.05
9	SO_4_	1.003	0.522	31.42	185.32	does not exist	does not exist	concave parabola	9.38	319.50
10	NO_3_	1.003	0.832	does not exist	71.45	does not exist	does not exist	concave parabola	1.33	227.09

Note: *s_r_*—coefficient of sensitivity; R^2^—coefficient of determination; LL—lower limit; UL—upper limit; * minimum—maximum contents of chemical elements/parameters in groundwater of the Slovak Republic (units in mg·L^−1^, Ca + Mg in mmol·L^−1^).

**Table 6:** Mean values for HI in selected areas of the Slovak Republic.

Geological Unit/District	1	8	Krupina	Bardejov	SR
Health Indicator	*n* = 727	*n* = 309	*n* = 36	*n* = 86	
LE	73.69	71.11	69.95	74.07	72.60
PYLL100	3874.38	4586.18	5609.07	3140.73	4033.00
ReC00-C97	177.99	236.28	243.23	175.32	212.79
ReI00-I99	463.32	638.78	889.20	492.82	531.05
ReJ00-J99	54.42	81.98	81.11	26.62	58.08
ReK00-K93	34.22	66.88	75.68	25.39	45.83
SMRC00-97	95.03	102.91	99.73	91.20	100
SMRI00-I99	100.03	108.50	131.06	100.71	100
SMRJ00-J99	109.39	126.34	116.33	50.50	100
SMRK00-K93	84.31	130.61	150.20	62.63	100
PYLLC00-C97	909.88	1097.32	1121.6	808.8	1005.20
PYLLI00-I99	831.99	1050.95	1518.2	779.9	866.19
PYLLJ00-J99	229.74	202.67	259.2	231.1	172.69
PYLLK00-K93	287.97	491.26	693.29	211.84	334.8

Note: 1—Paleogene Flysch; 8—Neovolcanic rocks; SR—mean for the Slovak Republic; *n* = number of municipalities in evaluated geological unit/district.

**Table 7:** Mean values for selected chemical elements and parameters in groundwater in selected areas of the Slovak Republic.

Geological Unit/District	1	8	Krupina	Bardejov	SR
Parameter	*n* = 727	*n* = 309	*n* = 36	*n* = 86	
TDS (mg·L^−1^)	524.64	439.73	362.34	484.79	629.75
Ca + Mg (mmol·L^−1^)	3.02	2.11	1.58	2.75	3.50
Na (mg·L^−1^)	12.74	16.09	13.12	10.34	20.34
Ca (mg·L^−1^)	88.53	56.13	42.01	80.75	93.56
Mg (mg·L^−1^)	19.67	17.14	12.96	17.98	28.29
Cl (mg·L^−1^)	17.14	21.66	13.81	13.77	32.96
SO_4_ (mg·L^−1^)	62.72	49.70	22.42	44.96	79.32
NO_3_ (mg·L^−1^)	16.19	26.44	16.49	14.84	38.76
HCO_3_ (mg·L^−1^)	287.65	191.51	174.23	282.12	303.85
As (mg·L^−1^)	0.00079	0.00241	0.0018	0.00114	0.00192
Se (mg·L^−1^)	0.00068	0.00086	0.0006	0.00068	0.00097
Pb (mg·L^−1^)	0.00125	0.00134	0.0018	0.00094	0.00136

Note: 1—Paleogene Flysch; 8—Neovolcanic rocks; SR—mean for the Slovak Republic; *n* = number of municipalities in evaluated geological unit/district.

**Table 8:** Derived limit values for Ca, Mg contents and water hardness for single HI.

Health Indicator	Order	Element	Limit Content		Optimal Content		Contents *	
			LL	UL	LL	UL	Min	Max
LE	1	Ca + Mg	2.98	does not exist	not defined	does not exist	0.35	7.97
	2	Ca	73.95	172.21	not defined	not defined	9.83	201.01
	3	Mg	18.13	does not exist	not defined	does not exist	2.45	97.75
PYLL100	1	Ca + Mg	2.87	6.67	3.21	6.33	0.35	7.97
	2	Ca	79.40	169.74	87.05	162.09	9.83	201.01
	3	Mg	20.44	83.24	33.82	69.87	2.45	97.75
ReC00-C97	3	Ca + Mg	1.73	5.85	2.23	5.34	0.35	7.97
	4	Ca	60.56	196.84	91.18	166.21	9.83	201.01
	8	Mg	25.66	35.83	12.72	48.77	2.45	97.75
SMRC00-C97	2	Ca + Mg	does not exist	4.17	does not exist	does not exist	0.35	7.97
	3	Ca	104.07	does not exist	does not exist	does not exist	9.83	201.01
	1	Mg	does not exist	33.50	does not exist	does not exist	2.45	97.75
PYLLC00-C97	1	Ca + Mg	not defined	not defined	not defined	not defined	0.35	7.97
	3	Ca	93.17	194.91	106.52	181.56	9.83	201.01
	4	Mg	not defined	not defined	not defined	not defined	2.45	97.75
ReI00-I99	1	Ca + Mg	2.90	9.10	4.40	7.60	0.35	7.97
	2	Ca	does not exist	89.40	does not exist	does not exist	9.83	201.01
	3	Mg	24.30	95.80	42.00	78.10	2.45	97.75
SMRI00-I99	1	Ca + Mg	not defined	not defined	not defined	not defined	0.35	7.97
	2	Ca	not defined	not defined	not defined	not defined	9.83	201.01
	3	Mg	does not exist	65.85	does not exist	does not exist	2.45	97.75
PYLLI00-I99	1	Ca + Mg	5.70	8.88	5.73	8.85	0.35	7.97
	3	Ca	150.76	does not exist	164.04	does not exist	9.83	201.01
	3	Mg	56.20	82.78	56.20	82.78	2.45	97.75
ReJ00-J99	1	Ca + Mg	3.20	11.67	5.88	8.99	0.35	7.97
	2	Ca	93.08	does not exist	does not exist	does not exist	9.83	201.01
	3	Mg	28.63	does not exist	83.99	120.05	2.45	97.75
SMRJ00-J99	6	Ca + Mg	3.27	does not exist	does not exist	does not exist	0.35	7.97
	3	Ca	90.03	does not exist	does not exist	does not exist	9.83	201.01
	4	Mg	25.81	does not exist	does not exist	does not exist	2.45	97.75
PYLLJ00-J99	4	Ca + Mg	does not exist	4.06	does not exist	does not exist	0.35	7.97
	2	Ca	does not exist	121.18	does not exist	does not exist	9.83	201.01
	3	Mg	does not exist	47.63	does not exist	does not exist	2.45	97.75
ReK00-K93	1	Ca + Mg	does not exist	4.08	0.41	3.53	0.35	7.97
	4	Ca	17.74	127.58	35.14	110.18	9.83	201.01
	7	Mg	does not exist	33.54	does not exist	10.65	2.45	97.75
SMRK00-K93	1	Ca + Mg	0.99	2.16	0.99	2.16	0.35	7.97
	2	Ca	not defined	not defined	not defined	not defined	9.83	201.01
	3	Mg	does not exist	29.67	does not exist	does not exist	2.45	97.75
PYLLK00-K93	1	Ca + Mg	does not exist	4.84	0.73	3.84	0.35	7.97
	2	Ca	17.58	173.05	57.80	132.83	9.83	201.01
	3	Mg	does not exist	37.27	does not exist	does not exist	2.45	97.75
Mean values		Ca + Mg	2.95	6.15	2.95	5.83	0.35	7.97
		Ca	78.03	155.61	89.61	152.29	9.83	201.01
		Mg	28.45	54.51	48.33	79.91	2.45	97.75
Limit values defined by Slovak guideline for drinking water [15]		*Ca* > *30* mg·L^−1^		*Mg 10–30* mg·L^−1^		*Ca* + *Mg 1.1–5.0* mmol·L^−1^		

Note: * Minimum and maximum groundwater contents in the Slovak Republic.

**Table 9:** Proposed limit values for groundwater used for drinking water public supply.

Parameter	Recommended Levels
Ca + Mg	2–5 mmol·L^−1^
Ca	50–180 mg·L^−1^
Mg	25–50 mg·L^−1^

**Table 10:** List of selected socio-economic, health-care and lifestyle characteristics for Krupina and Bardejov districts compared with the Slovak Republic (adapted from [28]).

Socio-Economic Characteristics ^a^	Krupina	Bardejov	SR
Level of registered unemployment (% of population)	16.95	19.6	*12.29*
Average nominal monthly salary in Euro	694	614	*957*
Rate of gypsy nationality (% of population)	2.1–4	4.1–8	*2*
Health-care characteristics ^b^			
No. of physicians posts per 10,000 population—adults (age 18+ years)	4.36	3.40	*4.32*
No. of physicians posts per 10,000 population—children and adolescents (age 0–17 years)	6.86	7.44	*9.87*
No. of vaccinated children and adolescents (age 0–17 years)	98.23	98.1	*97.9*
Lifestyle characteristics ^c,d^			
Regular physical activity in average (% of population)	45	39.5	*58.5*
Regular eating habits (% of population)	75	49	*68*
Smoking (% of population)	25	43	*19.5*
Excessive alcohol intake (% of population)	9.8	11	*6.8*

Note: ^a^ Statistical office of the Slovak Republic [39]; ^b^ NHIC—National Health Information Center [68]; ^c^ Data source for Krupina district: [69]; ^d^ Data source for Bardejov district and the Slovak Republic: EHES—European Health Examination Survey [70], SR—Slovak Republic.
